# Prophylactic Tranexamic Acid as Prevention for Early Postoperative Bleeding after Roux-en-Y Gastric Bypass

**DOI:** 10.1007/s11695-026-08658-6

**Published:** 2026-04-16

**Authors:** João Victor Vecchi Ferri, Marcela Scardua Cocicov, Vitor Mamoru Haida, José Alfredo Sadowski, Wagner Herbert Sobottka, Gustavo Rodrigues Alves Castro, João Caetano Dallegrave Marchesini, Alexandre Coutinho Teixeira de Freitas

**Affiliations:** 1 Clinical Hospital Complex of the Federal University of Paraná, Curitiba, Paraná, Brazil; 2 São Marcelino Champagnat Hospital, Curitiba, Paraná, Brazil; 3 Sugisawa Hospital, Curitiba, Paraná, Brazil

**Keywords:** Roux-en-Y gastric bypass, Tranexamic acid, Obesity, Postoperative hemorrhage, Thromboembolism, Bariatric surgery

## Abstract

**Background:**

Postoperative bleeding (POB) after Roux-en-Y gastric bypass (RYGB) occurs in up to 4.1% of cases and is associated with increased 30-day mortality, transfusions, and reinterventions.

**Methods:**

This study evaluated whether tranexamic acid (TXA) administered during anesthetic induction reduces clinical POB within 48 h after primary laparoscopic RYGB. Secondary outcomes included risk factors for POB, thromboembolic events (TEE), and mortality. In this mixed cohort study, consecutive patients undergoing RYGB between 2020 and 2025 were divided according to systematic TXA use.

**Results:**

Among 678 patients, 347 (51.2%) received TXA. This group’s mean age was 37 years and their mean BMI 40.4 kg/m². POB occurred in 21 patients (3.1%). TXA significantly reduced POB risk (OR 4.25; *p* = 0.007). Being of male sex increased POB risk (OR 2.59; *p* = 0.031). No differences in TEE or mortality were observed.

**Conclusions:**

TXA reduced POB risk by 70–78%. It was also noted that male sex was an independent risk factor.

## Key Points

Early POB after laparoscopic RYGB occurred in 3.1%.

TXA reduced the odds of bleeding by 70–78%.

Male sex was an independent risk factor for bleeding.

TXA did not increase the risk of TEE.

## Introduction

Postoperative bleeding (POB) has an incidence of 0.6 to 4.1% after Roux-en-Y gastric bypass (RYGB) [1–6]. It also increases mortality, need for blood transfusions, reinterventions and length of hospital stay [7, 8]. Tranexamic acid (TXA), a synthetic lysine analogue and potent antifibrinolytic agent, inhibits fibrinolysis by preventing plasminogen binding to fibrin, thereby promoting hemostasis [9–11], as corroborated by its use in orthopedic, gynecologic, obstetric, cardiac, and reconstructive procedures [12–15].

However, evidence regarding the efficacy and safety of TXA in RYGB remains limited. This study aimed to assess the impact of TXA on reducing POB after laparoscopic RYGB and to evaluate potential adverse outcomes, including thromboembolic events (TEE) and mortality.

## Materials and Methods

### Study Design and Participants

This cohort study included consecutive patients with obesity (≥ 16 years) who underwent laparoscopic RYGB between July 2020 and July 2025 at a private hospital. Sample size was defined by eligible patients included retrospectively and prospectively. The data was obtained from electronic medical records and surgeons’ personal databases.

Demographic variables included age and sex. Preoperative variables recorded included the following: BMI, ASA score, associated comorbid conditions (diabetes, hypertension, obstructive sleep apnea, smoking, and heart disease), recent use of specific medications (NSAIDs, antiplatelet agents or anticoagulant agents). It was also recorded if the patients had undergone an intragastric balloon placement (IGB) over the previous year or any history of hepatic steatosis, gastritis and *Helicobacter pylori* (*H. pylori*) infection, or extended postoperative prophylaxis with low-molecular-weight heparin (LMWH). All patients received *H. pylori* eradication therapy before surgery.

Exclusion criteria were robotic or open surgery, use of powered staplers, revisional procedures, major concomitant operations, previous gastric surgery or endosutures, and missing data. Minor associated procedures were not excluded (e.g. hiatoplasty, cholecystectomy). When ASA score was unavailable, the most recent classification was applied. Written informed consent was obtained from all prospectively included patients.

### RYGB Technique

All procedures were performed laparoscopically by the same team using a standardized technique. A 40–50 mL gastric pouch was created, with alimentary and biliopancreatic limbs of 100 cm and 120–150 cm, respectively, using an antecolic approach. Gastrojejunal and enteroenteric anastomoses were constructed using a manual linear stapler, mainly with Tri-Staple™ technology, and enterotomies sutured with 3 − 0 polydioxanone in two layers and one layer, respectively. Mesenteric defects were routinely closed, methylene blue leak testing was performed, and drains or anastomotic reinforcements were not routinely applied.

### Administration of TXA and Prevention of TEE

From June 2022, TXA was systematically administered, allowing division into two groups: TXA and non-TXA. A single intravenous dose of 1,000 mg was given at anesthetic induction. TXA was withheld in patients with prior or recent thromboembolic events.

All patients used intermittent pneumatic compression intraoperatively and received postoperative LMWH (40 mg daily) after surgery until discharge. Early ambulation and respiratory physiotherapy were encouraged. After discharge (24–36 h), extended LMWH for 10–14 days was prescribed at the discretion of the surgeon, and elastic stockings were recommended.

### Postoperative Evaluation

Outcomes included postoperative bleeding (POB) within 48 h, thromboembolic events and mortality within 30 days. POB was defined as hemorrhagic manifestation, whether from the upper or lower gastrointestinal tract, abdominal wall, or intraperitoneal bleeding. Residual blood streaks in the immediate postoperative period were not considered bleeding. Hematimetric indices were not routinely tested, only in cases of bleeding, which made comparison between groups unfeasible. Bleeding site, transfusion requirement, and reintervention were recorded. Extended (10–14 days) versus in-hospital LMWH (36 h) use was analyzed as a secondary subgroup for thromboembolic risk.

### Statistical Analysis

All data in this study was analyzed using Stata/SE v14.1 (StataCorp, College Station, TX, USA). Continuous variables were expressed as mean ± standard deviation or median (interquartile range), and categorical variables as frequencies (%). Comparisons used Student’s *t*-test, chi-square, or Fisher’s exact test. The association between TXA use and bleeding was assessed using Firth penalized logistic regression due to the low number of events. Odds ratios (OR) with 95% confidence intervals were calculated, and multivariate models adjusted for potential confounders. Statistical significance was designated by a p value < 0.05.

## Results

During the study period, 900 patients underwent laparoscopic RYGB. Then, after taking into account any exclusions, 678 were included in the final analysis. Of these 678 patients, 347 of them (51.2%) received TXA. TXA was withheld in some patients due to thrombophilia or recent cerebral venous thrombosis.

### Baseline Characteristics

Mean age was 37 years and mean BMI was 40.4 kg/m²; 520 patients (76.7%) were female. Most patients were classified as ASA II (75.2%). The most prevalent comorbidities were hepatic steatosis (67.0%), hypertension (34.1%), and sleep apnea (20.9%). Gastritis was present in 70.2% and *H. pylori* in 23.3% of patients (Table 1).

**Table 1:** Baseline demographic and preoperative factors among non-TXA and TXA groups

Variable	Total *N* (%) = 678 (100)	No TXA *N* (%) = 331 (48.8)	TXA *N* (%) = 347 (51.2)	p**
Age, years*	37.0 ± 10.4	36.8 ± 10.7	37.2 ± 10.2	0.629
BMI, kg/m^2^*	40.4 ± 4.9	40.5 ± 4.7	40.4 ± 5.1	0.943
Sex				
Female	520 (76.7)	245 (74)	275 (79.3)	0.122
Male	158 (23.3)	86 (26)	72 (20.7)	
ASA				
I	13 (1.9)	10 (3)	3 (0.9)	< 0.001
II	510 (75.2)	272 (82.2)	238 (68.6)	
III	155 (22.9)	49 (14.8)	106 (30.5)	
Diabetes	88 (13)	53 (16)	35 (10.1)	0.023
Sleep apnea	141 (20.9)	64 (19.5)	77 (22.2)	0.396
Hipertension	231 (34.1)	115 (34.7)	116 (33.4)	0.746
Smoking	44 (6.5)	22 (6.6)	22 (6.3)	0.878
Cardiopathy	11 (1.6)	6 (1.8)	5 (1.4)	0.768
NSAID	4 (0.6)	3 (0.9)	1 (0.3)	0.362
Antiplatelet	11 (1.6)	7 (2.1)	4 (1.2)	0.374
Anticoagulant	3 (0.4)	2 (0.6)	1 (0.3)	0.616
IGB	6 (0.9)	4 (1.2)	2 (0.6)	0.441
Hepatic steatosis	444 (67)	208 (65.8)	236 (68)	0.564
Gastritis	467 (70.2)	239 (75.2)	228 (65.7)	0.009
H. pylori	154 (23.3)	86 (27.3)	68 (19.7)	0.027
LMWH	214 (31.6)	67 (20.2)	147 (42.4)	< 0.001
TEE	1 (0.1)	0 (0)	1 (0.3)	1
Death	0 (0)	0 (0)	0 (0)	-

* Mean ± standard deviation

** Student’s t-test for independent samples (quantitative variables); Fisher’s exact test or Chi-square test (categorical variables); *p* < 0.05

Abbreviations: *ASA* American Society of Anesthesiologists score, *BMI* body mass index, *IGB* intragastric balloon, *LMWH* extended prophylaxis with low molecular weight heparin, *NSAID* nonsteroidal anti-inflammatory drug, *TEE* thromboembolic event

### Early Postoperative Bleeding (POB)

POB occurred in 21 patients (3.1%). Bleeding sites included lower gastrointestinal tract (76.1%), abdominal wall (9.5%), intracavitary (9.5%), and upper gastrointestinal tract (4.7%). Most cases were managed conservatively (85.7%); blood transfusion was required in 47.6%, and 14.2% underwent surgical exploration. No endoscopic or angiographic interventions were performed.

TXA significantly reduced bleeding risk (OR 4.25; *p* = 0.007) and remained independently protective in multivariate models (OR 3.72–4.47). Male individuals had a significantly worse clinical profile, such as higher BMI, higher ASA class, and greater prevalence of comorbidities (Table 2). Male sex was also associated with increased bleeding risk (OR 2.59; *p* = 0.031), even when adjusted for other variables through multivariate analysis, showing an independent strength of association with bleeding. No other variables were significantly associated with POB (Table 3).

**Table 2:** Data analysis according to sex

Variable	Female *N* (%) = 520 (76.7)	Male *N* (%) = 158 (23.3)	p**
Age, years*	36.6 ± 10.6	38.4 ± 9.9	0.052
BMI (kg/m2)*	39.9 ± 4.7	42.4 ± 5.1	< 0.001
ASA			
I	11 (2.1)	2 (1.3)	
II	405 (77.9)	105 (66.5)	
III	104 (20)	51 (32.3)	0.005
Diabetes	53 (10.2)	35 (22.2)	< 0.001
Sleep apnea	74 (14.3)	67 (42.4)	< 0.001
Hipertension	159 (30.6)	72 (45.6)	< 0.001
Smoking	27 (5.2)	17 (10.8)	0.017
Cardiopathy	8 (1.5)	3 (1.9)	0.724
NSAID	1 (0.2)	3 (1.9)	0.041
Antiplatelet	7 (1.3)	4 (2.5)	0.293
Anticoagulant	0 (0)	3 (1.9)	0.012
IGB	6 (1.2)	0 (0)	0.345
Hepatic steatosis	308 (60.7)	136 (87.2)	< 0,001
Gastritis	344 (67.6)	123 (78.8)	0.007
H. pylori	115 (22.9)	39 (24.8)	0.666
LMWH	161 (31)	53 (33.5)	0.558
TEE	1 (0.2)	0 (0)	1

* Mean ± standard deviation

** Student’s t-test for independent samples (quantitative variables); Fisher’s exact test for categorical variables; *p* < 0.05

Abbreviations: *ASA* American Society of Anesthesiologists score, *BMI* body mass index, *IGB* intragastric balloon, *NSAID* nonsteroidal anti-inflammatory drug, *LMWH* extended prophylaxis with low molecular weight heparin, *TEE* thromboembolic event

**Table 3:** Data analysis in patients with and without early postoperative bleeding

Variable	No bleeding *N* (%) = 657 (96.9)	Bleeding *N* (%) = 21 (3.1)	p**	OR (95% CI)
TXA	314 (94.9)	17 (5.1)	0.007	4.25 (1.49–12.1)
Age, years*	37.0 ± 10.5	39.0 ± 8.5	0.354	1.02 (0.98–1.06)
Sex				
Female	508 (97.7)	12 (2.3)		
Male	149 (94.3)	9 (5.7)	0.031	2.59 (1.09–6.13)
ASA				
I	12 (92.3)	1 (7.7)		
II	492 (96.5)	18 (3.5)	0.195	0.31 (0.05–1.81)
III	153 (98.7)	2 (1.3)	0.063	0.14 (0.02–1.11)
BMI (kg/m^2^)*	40.5 ± 4.9	39.7 ± 4.0	0.577	0.97 (0.89–1.07)
Diabetes	87 (98.9)	1 (1.1)	0.386	0.48 (0.09–2.54)
Sleep apnea	135 (95.7)	6 (4.3)	0.320	1,61 (0.63–4.10)
Hipertension	222 (96.1)	9 (3.9)	0.365	1.49 (0.63–3.51)
Smoking	43 (97.7)	1 (2.3)	0.969	1.03 (0.19–5.58)
Hepatic steatosis	427 (96.2)	17 (3.8)	0.116	2.53 (0.79–8.07)
Gastritis	453 (97)	14 (3)	0.909	0.95 (0.37–2.43)
H. pylori	149 (96.8)	5 (3.2)	0.760	1.17 (0.43–3.14)
LMWH	211 (98.6)	3 (1.4)	0.097	0.35 (0.10–1.21)
Cardiopathy	11 (100)	0 (0)	1^†^	-
NSAID	3 (75)	1 (25)	0.119^†^	-
Antiplatelet	11 (100)	0 (0)	1^†^	-
Anticoagulant	2 (66.7)	1 (33.3)	0.090^†^	-
IGB	6 (100)	0 (0)	1^†^	-

* Mean ± standard deviation

** Penalized Logistic Regression Model (Firth), significance assessed by the penalized likelihood ratio (PLR) test, *p* < 0.05

^†^ Fisher’s exact test, *p* < 0.05

Abbreviations: *BMI* body mass index, *IGB* intragastric balloon, *LMWH* extended prophylaxis with low molecular weight heparin, *NSAID* nonsteroidal anti-inflammatory drug, *OR* odds ratio, *TXA* tranexamic acid;

### Secondary Outcomes

One patient developed a thromboembolic event (lower-limb deep vein thrombosis) on postoperative day 7 in the TXA group, associated with early resumption of oral contraceptives. No deaths occurred.

## Discussion

POB is associated with longer hospital stay, increased 30-day mortality, and higher rates of postoperative complications, including venous thromboembolism (VTE) [16, 17]. Reoperations for hemostasis after RYGB range from 0.8% to 2.5% [18].

Among bleeding-related factors, two deserve emphasis. Electric staplers appear to reduce POB and transfusion requirements, likely due to greater stability during tissue compression [19], and were therefore excluded from this study to minimize confounding factors. Conversely, prior IGB placement increases POB risk up to threefold in metabolic and bariatric surgery (MBS), particularly RYGB, possibly due to chronic inflammation, hypertrophy, and fibrosis of the gastric wall [20, 21].

Evidence on TXA use in MBS remains limited and is largely derived from sleeve gastrectomy (SG). Klaasen reported reduced reoperation rates without increased TEE when TXA was used therapeutically after bleeding [22]. Prophylactic TXA reduced intraoperative bleeding, operative time, and hospital stay in SG without increasing TEE risk [23]. A meta-analysis of 475 SG patients showed a significant reduction in POB (OR 0.40; *p* = 0.002), despite heterogeneity in dosing and timing, with no increase in TEE or mortality [24].

Lo et al. (2024) evaluated POB up to 30 days after RYGB. Groups that received TXA showed a smaller drop in hemoglobin (*p* = 0.011), and omitting enoxaparin did not affect this outcome. A higher number of staple firings (eight vs. seven) was associated with increased bleeding risk (*p* = 0.001), likely reflecting a larger exposed tissue area at risk for adverse events [6]. A recent meta-analysis of SG and RYGB confirmed reductions in POB, operative time, and hospitalization, with no effect on TEE or mortality [25]. Larger retrospective analyses found no differences in transfusions, reoperations, or TEE across bariatric procedures [26], while TXA reduced only minor bleeding-related complications and length of stay without increasing VTE risk after SG in another study [27].

Uncertainty remains regarding optimal TXA dosing and timing. Randomized trials showed reduced POB with TXA at anesthetic induction or surgery end, with fewer intraoperative bleeding points when administered at induction and no increase in TEE [28]. Pre-extubation administration also reduced bleeding without increasing VTE risk in another study [29]. Meta-analyses suggest no additional benefit from doses above 14–15 mg/kg [30, 31].

The low incidence of VTE after MBS (0.34–0.5% at 30 days) may limit statistical power to detect differences in our study [32, 33]. Large meta-analyses across surgical settings, including 125,550 patients, demonstrated no increased TEE risk with TXA and reduced bleeding-related mortality [34], despite concerns raised in specific contexts, such as high-dose infusions in trauma setting, gastrointestinal bleeding [35–37].

The POISE-3 trial showed that TXA reduced bleeding-related events by 25%, with a small absolute increase in cardiovascular outcomes, highlighting the need for individualized risk–benefit assessment [38]. In a POISE-3 subgroup analysis limited to general surgery (*n* = 3,260), however, the same TXA regimen significantly reduced severe postoperative bleeding (8.0% vs. 10.5%, *p* = 0.01) without differences in cardiovascular safety [39].

Bleeding often leads to interruption of thromboprophylaxis, increasing VTE risk in patients with morbid obesity. Transfusions and postoperative inflammation further contribute to a prothrombotic state [17, 40]. Preventing POB may therefore indirectly reduce VTE risk.

Male sex has consistently been associated with higher POB risk after RYGB. Meta-analyses and large cohort studies identified male sex as an independent risk factor for bleeding, complications, and 30-day mortality, even after adjustment for BMI, age, and comorbidities [41–43]. Potential mechanisms include greater visceral fat, larger liver volume, reduced intra-abdominal space, and sex-related differences in inflammatory and vascular profiles [44, 45].

### Final Considerations

This study is limited by its mixed, non-randomized design with a retrospective component and its inherent risk of bias. Only clinically significant bleeding was assessed, as hematimetric parameters were not routinely measured to detect asymptomatic events.

In conclusion, early POB after RYGB occurred in 3.1% of patients. TXA showed an association with a 70–78% reduction in bleeding across all models. Male sex showed an increased independent association with the risk of bleeding, while TXA use was not associated with an increased risk of thromboembolic events, although the low number of events limits this conclusion.

## Data Availability

All data supporting the findings of this study are available within the paper and its Supplementary Information, called blinded_data.

## References

[1] Schauer PR, Ikramuddin S, Gourash W. Outcomes after laparoscopic Roux-en-Y gastric bypass for morbid obesity. Ann Surg. 2000;232:515–29. 10998650 10.1097/00000658-200010000-00007 PMC1421184

[2] Podnos YD, Jimenex JC, Wilson SE, Stevens CM, Nguyen NT. Complications after laparoscopic gastric bypass: a review of 3464 cases. Arch Surg. 2003;9:957–61. 10.1001/archsurg.138.9.957 12963651

[3] Nguyen NT, Rivers R, Wolfe BM. Early gastrointestinal hemorrhage after laparoscopic gastric bypass. Obes Surg. 2003;1:62–5. 10.1381/096089203321136601 12630615

[4] Mehran A, Szomstein S, Zundel N, Rosenthal R. Management of acute bleeding after laparoscopic Roux-en-Y gastric bypass. Obes Surg. 2003;6:842–7. 10.1381/096089203322618623 14738667

[5] Pereira A, Santos RF, Costa-Pinho A, Silva A, Nogueiro J, Carneiro S, Lima-da-Costa E, Santos-Sousa H, Preto J. Early Postoperative Bleeding After Laparoscopic Roux-En-Y Gastric Bypass: a Single Center Analysis. Obes Surg. 2022;32(6):1902–8. 10.1007/s11695-022-05973-6. 35201569 10.1007/s11695-022-05973-6

[6] Lo HC, Hsu SC, Soong RS, Huang SK. Unraveling Postoperative Bleeding Dynamics in Laparoscopic Roux-en-Y Gastric Bypass: Insights from a Single-Center Tranexamic Acid Study. Obes Surg. 2024;34(8):3012–20. 10.1007/s11695-024-07411-1. 39037676 10.1007/s11695-024-07411-1

[7] Spence J, LeManach Y, Chan MTV, et al. Vascular Events in Noncardiac Surgery Patients Cohort Evaluation (VISION) Study Investigators. Association between complications and death within 30 days after noncardiac surgery. CMAJ. 2019;191(30):E830–7. 10.1503/cmaj.190221. 31358597 10.1503/cmaj.190221 PMC6663503

[8] Smilowitz NR, Ruetzler K, Berger JS. Perioperative bleeding and outcomes after noncardiac surgery. Am Heart J. 2023;260:26–33. 10.1016/j.ahj.2023.02.008. 36801264 10.1016/j.ahj.2023.02.008 PMC10164115

[9] Cai J, Ribkoff J, Olson S, et al. The many roles of tranexamic acid: an overview of the clinical indications for TXA in medical and surgical patients. Eur J Haematol. 2020;104(2):79–87. 10.1111/ejh.13348. 31729076 10.1111/ejh.13348 PMC7023891

[10] Chauncey JM, Patel P, Tranexamic Acid. [Updated 2025 Apr 26]. In: StatPearls [Internet]. Treasure Island (FL): StatPearls Publishing; 2025 Jan-. Available from: https://www.ncbi.nlm.nih.gov/books/NBK532909/30422504

[11] Astedt B. Clinical pharmacology of tranexamic acid. Scand J Gastroenterol Suppl. 1987;137:22–5. PMID: 3321402. 3321402

[12] Henry DA, Carless PA, Moxey AJ, O’Connell D, Stokes BJ, Fergusson DA et al. Anti-fibrinolytic use for minimising perioperative allogeneic blood transfusion. Cochrane Db Syst Rev. 2011;(3). 10.1002/14651858.CD001886.pub2 17943760

[13] Oremus K. Tranexamic acid for the reduction of blood loss in total knee arthroplasty. Ann Transl Med. 2015;3(Suppl 1):S40. 26046088 10.3978/j.issn.2305-5839.2015.03.35 PMC4437933

[14] WOMAN Trial Collaborators. Effect of early tranexamic acid administration on mortality, hysterectomy, and other morbidities in women with post-partum haemorrhage (WOMAN): an international, randomised, double-blind, placebo-controlled trial. Lancet. 2017;389(10084):2105–16. 10.1016/S0140-6736(17)30638-4. 28456509 10.1016/S0140-6736(17)30638-4 PMC5446563

[15] Brown S, Brown T, Rohrich RJ. Clinical Applications of Tranexamic Acid in Plastic and Reconstructive Surgery. Plast Reconstr Surg. 2024;154(6):e1253–63. 10.1097/PRS.0000000000011288. 10.1097/PRS.0000000000011288 38196097

[16] Zafar SN, Miller K, Felton J, Wise ES, Kligman M. Postoperative bleeding after laparoscopic Roux en Y gastric bypass: predictors and consequences. Surg Endosc. 2019;33(1):272–80. 10.1007/s00464-018-6365-z. 30232617 10.1007/s00464-018-6365-z

[17] Nielsen AW, Helm MC, Kindel T, Higgins R, Lak K, Helmen ZM, Gould JC. Perioperative bleeding and blood transfusion are major risk factors for venous thromboembolism following bariatric surgery. Surg Endosc. 2018;32(5):2488–95. 10.1007/s00464-017-5951-9. 29101558 10.1007/s00464-017-5951-9

[18] Augustin T, Aminian A, Romero-Talamás H, Rogula T, Schauer PR, Brethauer SA. Reoperative Surgery for Management of Early Complications After Gastric Bypass. Obes Surg. 2016;26(2):345–9. 10.1007/s11695-015-1767-7. 26140855 10.1007/s11695-015-1767-7

[19] Roy S, Yoo A, Yadalam S, Fegelman EJ, Kalsekar I, Johnston SS. Comparison of economic and clinical outcomes between patients undergoing laparoscopic bariatric surgery with powered versus manual endoscopic surgical staplers. J Med Econ. 2017;20(4):423–33. 10.1080/13696998.2017. 28270023 10.1080/13696998.2017.1296453

[20] Golzarand M, Toolabi K, Parsaei R. Prediction Factors of Early Postoperative Bleeding after Bariatric Surgery. Obes Surg. 2022;32(7):1–8. 10.1007/s11695-022-06059-z. 35474043 10.1007/s11695-022-06059-z

[21] Patrzyk M, Sonke J, Glitsch A, Kessler R, Steveling A, Lünse S, Partecke LI, Heidecke CD, Kessler W. Gastric Balloon Implantation as Part of Morbid Adiposity Therapy Changes the Structure of the Stomach Wall. Visc Med. 2021;37(5):418–25. 10.1159/000514264. 34722725 10.1159/000514264 PMC8543320

[22] Klaassen RA, Selles CA, van den Berg JW, Poelman MM, van der Harst E. Tranexamic acid therapy for postoperative bleeding after bariatric surgery. BMC Obes. 2018;5:36. 10.1186/s40608-018-0213-5. 30524741 10.1186/s40608-018-0213-5 PMC6276262

[23] Lech P, Michalik M, Waczyński K, Osowiecka K, Dowgiałło-Gornowicz N. Effectiveness of prophylactic doses of tranexamic acid in reducing hemorrhagic events in sleeve gastrectomy. Langenbecks Arch Surg. 2022;407(7):2733–7. 10.1007/s00423-022-02630-5. 35920900 10.1007/s00423-022-02630-5 PMC9640392

[24] Mocanu V, Wilson H, Verhoeff K, Kung J, Walsh C, Koloszvari N, Neville A, Karmali S. Role of Tranexamic Acid (TXA) in Preventing Bleeding Following Sleeve Gastrectomy: a Systematic Review and Meta-analysis. Obes Surg. 2023;33(5):1571–9. 10.1007/s11695-023-06563-w. 36977890 10.1007/s11695-023-06563-w

[25] Lech GE, Vidotto LM, Sturmer CM, da Silveira CAB, Kasakewitch JPG, Lima DL, Zhou Y, Choi J, Camacho D, Moran-Atkin E. The Role of Tranexamic Acid (TXA) on Postoperative Bleeding in Bariatric Surgery: A Systematic Review and Meta-Analysis. Obes Surg. 2025;35(4):1504–12. 10.1007/s11695-025-07709-8. 40072741 10.1007/s11695-025-07709-8

[26] Lourie NEE, Kupietzky A, Maden A, Sharvit S, Ronen A, Umansky M, Mizrahi I, Mazeh H, Ben-Zvi D, Grinbaum R. The Effect of Preoperative Single-Dose Tranexamic Acid on Bleeding and Thromboembolic Events Following Bariatric Surgery. Obes Surg. 2025;28. 10.1007/s11695-025-07764-1. 10.1007/s11695-025-07764-1 40153243

[27] ‘t Hart JWH, Noordman BJ, Wijnand JMA, et al. Peroperative administration of tranexamic acid in sleeve gastrectomy to reduce hemorrhage: a double-blind randomized controlled trial. Surg Endosc. 2023;37(10):7455–63. 10.1007/s00464-023-10232-5. 37400687 10.1007/s00464-023-10232-5 PMC10520143

[28] Sermet M, Ozsoy MS. Effect of Tranexamic Acid on Postoperative Bleeding in Sleeve Gastrectomy: a Randomized Trial. Obes Surg. 2023;33(12):3962–70. 10.1007/s11695-023-06902-x. 37857939 10.1007/s11695-023-06902-x

[29] Hossain N, Kaur V, Mahran M, Quddus A, Mukhopadhyay S, Shah A, Agrawal S. Intra-operative Tranexamic Acid Administration Significantly Decreases Incidence of Postoperative Bleeding Without Increasing Venous Thromboembolism Risk After Laparoscopic Sleeve Gastrectomy: a Retrospective Cohort Study of Over 400 Patients. Obes Surg. 2024;34(2):396–401. 10.1007/s11695-023-07021-3. 38168716 10.1007/s11695-023-07021-3

[30] Ker K, Prieto-Merino D, Roberts I. Systematic review, meta-analysis and meta-regression of the effect of tranexamic acid on surgical blood loss. Br J Surg. 2013;100(10):1271–9. 10.1002/bjs.9193. 23839785 10.1002/bjs.9193

[31] Heyns M, Knight P, Steve AK, Yeung JK. A Single Preoperative Dose of Tranexamic Acid Reduces Perioperative Blood Loss: A Meta-analysis. Ann Surg. 2021;273(1):75–81. 10.1097/SLA.0000000000003793. 32224739 10.1097/SLA.0000000000003793

[32] Thereaux J, Lesuffleur T, Czernichow S, Basdevant A, Msika S, Nocca D, Millat B, Fagot-Campagna A. To What Extent Does Posthospital Discharge Chemoprophylaxis Prevent Venous Thromboembolism After Bariatric Surgery? Results From a Nationwide Cohort of More Than 110,000 Patients. Ann Surg. 2018;267(4):727–33. 10.1097/SLA.0000000000002285. 28475558 10.1097/SLA.0000000000002285

[33] El Ansari W, El-Menyar A, El-Ansari K, Al-Ansari A, Lock M. Cumulative Incidence of Venous Thromboembolic Events In-Hospital, and at 1, 3, 6, and 12 Months After Metabolic and Bariatric Surgery: Systematic Review of 87 Studies and Meta-analysis of 2,731,797 Patients. Obes Surg. 2024;34(6):2154–76. 10.1007/s11695-024-07184-7. 38602603 10.1007/s11695-024-07184-7 PMC11127857

[34] Taeuber I, Weibel S, Herrmann E, et al. Association of Intravenous Tranexamic Acid With Thromboembolic Events and Mortality: A Systematic Review, Meta-analysis, and Meta-regression. JAMA Surg. 2021;156(6):e210884. 10.1001/jamasurg.2021.0884. 33851983 10.1001/jamasurg.2021.0884 PMC8047805

[35] Johnston LR, Rodriguez CJ, Elster EA, et al. Evaluation of Military Use of Tranexamic Acid and Associated Thromboembolic Events. JAMA Surg. 2018;153(2):169–75. 10.1001/jamasurg.2017.3821. 29071337 10.1001/jamasurg.2017.3821 PMC5838717

[36] Roberts I, et al. Effects of a high-dose 24-h infusion of tranexamic acid on surgical bleeding and death due to bleeding in trauma patients. Lancet. 2020;396(10249):23–32. 10.1016/S0140-6736(20)30848-5.32622388

[37] HALT-IT Trial Collaborators. Effects of a high-dose 24-h infusion of tranexamic acid on death and thromboembolic events in patients with acute gastrointestinal bleeding (HALT-IT): an international randomised, double-blind, placebo-controlled trial. Lancet. 2020;395(10241):1927–36. 10.1016/S0140-6736(20)30848-5. 32563378 10.1016/S0140-6736(20)30848-5 PMC7306161

[38] Devereaux PJ, Marcucci M, Painter TW, et al. Tranexamic Acid in Patients Undergoing Noncardiac Surgery. N Engl J Med. 2022;386(21):1986–97. 10.1056/NEJMoa2201171. 35363452 10.1056/NEJMoa2201171

[39] Park LJ, Marcucci M, Ofori SN, et al. Safety and Efficacy of Tranexamic Acid in General Surgery. JAMA Surg. 2025;15. 10.1001/jamasurg.2024.6048. 10.1001/jamasurg.2024.6048 PMC11904705 39813061

[40] Albayati MA, Grover SP, Saha P, et al. Postsurgical Inflammation as a Causative Mechanism of Venous Thromboembolism. Semin Thromb Hemost. 2015;41(6):615–20. 10.1055/s-0035-1556726. 26276933 10.1055/s-0035-1556726

[41] Santos-Sousa H, Amorim-Cruz F, Nogueiro J, et al. Preoperative risk factors for early postoperative bleeding after Roux-en-Y gastric bypass surgery: a systematic review and meta-analysis. Langenbecks Arch Surg. 2024;409(1):163. 10.1007/s00423-024-03346-4. 38775865 10.1007/s00423-024-03346-4 PMC11111548

[42] Dayer-Jankechova A, Fournier P, Allemann P, Suter M. Complications After Laparoscopic Roux-en-Y Gastric Bypass in 1573 Consecutive Patients: Are There Predictors? Obes Surg. 2016;26(1):12–20. 10.1007/s11695-015-1752-1. 26058754 10.1007/s11695-015-1752-1

[43] Dugan N, Thompson KJ, Barbat S, Prasad T, McKillop IH, Maloney SR, Roberts A, Gersin KS, Kuwada TS, Nimeri A. Male gender is an independent risk factor for patients undergoing laparoscopic sleeve gastrectomy or Roux-en-Y gastric bypass: an MBSAQIP^®^ database analysis. Surg Endosc. 2020;34(8):3574–83. 10.1007/s00464-019-07106-0. 32072290 10.1007/s00464-019-07106-0 PMC7224103

[44] Palmer BF, Clegg DJ. The sexual dimorphism of obesity. Mol Cell Endocrinol. 2015;402:113–9. 10.1016/j.mce.2014.11.029. 25578600 10.1016/j.mce.2014.11.029 PMC4326001

[45] Blum A, Tamir S, Hazzan D, Podvitzky O, Sirchan R, Keinan-Boker L, Ben-Shushan RS, Blum N, Suliman LS, Geron N. Gender effect on vascular inflammation following bariatric surgery. Eur Cytokine Netw. 2012;23(4):154–7. 10.1684/ecn.2012.0318. 10.1684/ecn.2012.0318 23306174

